# High prevalence of hepatitis B infections in Burkina Faso (1996–2017): a systematic review with meta-analysis of epidemiological studies

**DOI:** 10.1186/s12889-018-5432-7

**Published:** 2018-04-25

**Authors:** Moussa Lingani, Tomoyuki Akita, Serge Ouoba, Armel Moumini Sanou, Aya Sugiyama, Zekiba Tarnagda, Masayuki Ohisa, Halidou Tinto, Shunji Mishiro, Junko Tanaka

**Affiliations:** 1 Unité de Recherche Clinique de Nanoro, Institut de Recherche en Sciences de la Santé, Nanoro, BP 218 Burkina Faso; 2 Department of Epidemiology, Infectious Disease Control and Prevention, Graduate School of Biomedical and Health Sciences, Hiroshima University, Hiroshima, Japan; 3 Unité des Maladies à potentiel épidémiques, Maladies émergentes et Zoonoses, Institut de Recherche en Sciences de la Santé, Bobo-Dioulasso, Burkina Faso; 4 Department of Medical Sciences, Toshiba General Hospital, Tokyo, Japan

**Keywords:** Hepatitis B, Viral infection, Burkina Faso, Epidemiology, Meta-analysis

## Abstract

**Background:**

Hepatitis B virus (HBV) infection was long considered an important public health concern in Burkina Faso and still represents a major cause of liver cancer and cirrhosis in the active population. To counter the problem, a national strategic plan was developed and adopted in July 2017 to coordinate viral hepatitis elimination’s efforts. However evidence to support its implementation remains scanty and scattered. The main purpose of this study was to summarize available information from per-reviewed articles published over the last two decades to accurately estimate the prevalence of HBV infection in Burkina Faso.

**Methods:**

We conducted a systematic search with meta-analysis of scientific articles using Science-Direct, Web-of-Science, PubMed/Medline, and Google Scholar. We systematically assessed all relevant publications that measured the prevalence of hepatitis B surface antigen and which were published between 1996 and 2017. We estimated the national HBV prevalence and its 95% confident interval. We subsequently adjusted the meta-analysis to possible sources of heterogeneity.

**Results:**

We retrieved and analyzed a total of 22 full text papers including 99,672 participants. The overall prevalence was 11.21%. The prevalence after adjustment were 9.41%, 11.11%, 11.73% and 12.61% in the general population, pregnant women, blood donors and HIV-positive persons respectively. The prevalence was higher before implementation of HBV universal vaccination and decreased from 12.80% between 1996 and 2001 to 11.11% between 2012 and 2017. The prevalence was also higher in rural area 17.35% than urban area 11.11%. The western regions were more affected with 12.69% than the central regions 10.57%. The prevalence was 14.66% in the boucle of Mouhoun region and 14.59 in the center-west region. Aggregate data were not available for the other regions.

**Conclusions:**

HBV has clearly an important burden in Burkina Faso as described by its high prevalence and this problem significantly challenges the national health care system. There is an urgent need for effective public health interventions to eliminate the problem. However, higher quality data are needed to produce reliable epidemiological estimates that will guide control efforts towards the achievement of the national strategic plan’s goals.

**Electronic supplementary material:**

The online version of this article (10.1186/s12889-018-5432-7) contains supplementary material, which is available to authorized users.

## Background

Viral hepatitis infections, including hepatitis B, represent a major public health problem worldwide and affect more people than HIV [1]. They are major cause of disability and mortality and represent the seventh leading cause of death worldwide [2]. Half of this burden is attributable to HBV, an important cause of chronic liver diseases particularly in Sub-Saharan Africa [3]. According to the WHO’s reports, more than 257 million people were chronically infected in 2015; among them 887,000 deaths due to liver cirrhosis and hepatocellular carcinoma (HCC) [4]. HCC is a very aggressive cancer with limited treatment options. It remains the commonest cancer among men and third among women in Africa where it represents the second leading cause of death by cancer following tobacco use [5, 6]. HBV infection’s burden remains high in Sub Saharan Africa with 65 million people chronically infected, and a prevalence rate between 6% and 20% [7, 8]. Mother-to-child transmission is the main route of contamination in Sub-Saharan Africa, and perinatal transmission is predominant [9]. Children born to hepatitis B e antigen (HBeAg) positive mother have 70–90% likelihood of perinatal infection, and up to 90% will evolve chronically as compared to only 5% in adulthood infections [10, 11].

Until recently, viral hepatitis has been widely ignored as an important public health priority despite its heavy burden. However since 2015, the United Nations has adopted a global resolution in its Agenda for Sustainable Development, which goal 3 calls for specific actions to eliminate viral hepatitis by 2030. Subsequently, in May 2016, the world health assembly adopted the first “Global Health Sector Strategy on Viral Hepatitis” with a vision to eliminate viral hepatitis by 2030 through coherent public health interventions [12]. The enormity of health losses due to viral hepatitis infections and the availability of effective tools, suggests there is an important opportunity to improve health at all levels [12].

Burkina Faso, a west African country, was listed among the high transmission countries define by a HBV infections prevalence in people above 8% [1, 8]. In Burkina Faso, while acute hepatitis B infections is costing 300 lives/year, liver cancer and cirrhosis of hepatitis B origin cause 800 and 1300 deaths/year respectively [13] and a general prevalence of about 14.5% of hepatitis B infection [14]. Studies conducted in the field tended to support this observation. Indeed, Nagalo et al. have reported HBsAg seroprevalence around 14.9% among blood donors in 2009 [15]. A voluntary testing conducted in 2014 by Tao et al. in the general population reported similar prevalence of 14.47% [16]. Meanwhile, Sangare et al.*,* also in 2009, have reported 11.4% among pregnant women [17]. A study conducted in Sub Saharan Africa in 2010 by Barth et al. which included HIV positive participants from Burkina Faso reported a prevalence between ten and 28% in the country [18]. If data on the epidemiology of hepatitis infection seems scanty, and the information available mainly from sentinel populations, it appears that Burkina Faso is a country of high transmission of HBV infection [3, 19, 20].

Since 2006, Burkina Faso has started HBV universal vaccination as part of the Expanded program on Immunization (EPI) without prior testing for HBV infection markers. And following the WHO recommendations; health authorities have adopted a strategic plan to control viral hepatitis over the period between 2017 and 2021 [21]. The key points of this strategic plan includes the development of publicly-funded screening and treatment centers, universal immunization for hepatitis B, the development and promotion of preventive activities, the reinforcement of the coordination, development of an effective supply chain for affordable medicines, development of an adequate communication system and also the establishment of surveillance system as well as the promotion of innovative research. This plan will allow a nationwide implementation of effective control measure with the involvement of all stakeholders and the promotion of policies that will reduce the burden of the disease throughout the country. In a context of limited resources, it appears critical to adequately allocate available resource to key intervention sectors that will generate the maximum impact in reducing the burden of the disease. Therefore, it appears fundamental to summarize already available information to improved understanding of the overall prevalence of the infection, its repartition over the country, and factors that sustain high transmission level as a first step toward the elimination program. For this purpose, we aim to conduct a systematic review to thoroughly summarize the available information towards answering the key question; how common is hepatitis B infection in Burkina Faso populations through a systematic analysis of peer-reviewed studies published between 1996 and 2017.

## Methods

### Study area

Burkina Faso is a landlocked country with a landmass of 274,000 km^2^ located in West Africa. The country shares common borders with six countries including Mali, Niger, Benin, Togo, Ghana and Ivory Coast. The climate is tropical with an alternating long dry season and a short rainy season. The country is organized in 13 regions. According to the population estimate extrapolated from the 2006 national census, the population was 19,034,397 inhabitants in 2016 of which 77.3% were in rural area, with farming as main occupation [22]. The population grows about 3.1% per year and women represent 51.7%. The birth rate is 46 per 1000, and the youth under 15 years old represent 46.4% of the population [20]. According to the health and demographic survey of 2010, the fertility rate is 6.0 per women. Death rate is 11.8‰ and life expectancy at birth is 57.5 years for females and 55.8 years for male. It is a low-income country with a GDP per capita USD 622.0 in 2012. Almost 40% of the population lived below the poverty line in 2014. The country was ranked 183/188 according the United Nation development program with an index of human development at 0.402 in 2015 [23]. This state of poverty is characterized by a limited access to basic health services and a high rate of malnutrition. More than 60 ethnic groups are reported with three main religious groups. The health system is organized in 13 health regions operationalized by 70 health districts. The doctor-patient-ratio in 2016 is 1:15836 [19]. In 2012, 12.4% of the national budget was allocated to health sector. Traditional therapies take an important part of the health system and represent a major part in the management of hepatitis B infection. The diseases of public health importance include malaria, acute respiratory infections, malnutrition, diarrheal diseases, HIV/AIDS, sexually transmitted infections, tuberculosis, leprosy, and tropical neglected diseases. In addition, the country is place of bacterial, measles and other epidemic diseases [19]. Even though viral hepatitis represents a major concern among population, it is not listed among country priority diseases.

### Literature search method

To identify studies, we have conducted a systematic database search of peer-reviewed literature addressing HBsAg prevalence by following the 2009 Preferred Reporting Items for Systematic Reviews and Meta-Analysis (PRISMA) statement guidelines [24]. A concept map was established using key words and medical subject headings (MeSH) including hepatitis B, hepatitis surface antigen, seroprevalence, Burkina Faso, and similar terms such HBV, HBsAg. A comprehensive search was carried out in PubMed/Medline, Google Scholar, Science Direct and the Web of Science with combination of MeSH terms and key words used in research equation with ‘OR’ and ‘AND’ logical operators. Detailed of such a search strategy for PubMed was “((hepatitis B[Mesh]) OR (hepatitis surface antigen[Mesh])) AND (seroprevalence[Mesh]) AND (Burkina Faso[Mesh])”. This search strategy was subsequently modified and adapted to other databases. Relevant papers identified and their reference lists manually assessed to identify additional articles. Duplicate entries were removed and all references were managed with a bibliographic management tool Mendeley Desktop version 1.17.10. The last search was conducted on August 15th, 2017. Study timespan covered period from January 1st, 1996 to August 15th, 2017. Search was limited to study conducted in human, and published either in French or in English.

### Inclusion/exclusion criteria

We assessed for eligibility, all peer-reviewed articles published between January 1st, 1996 and August 15th, 2017, which have reported HBsAg seroprevalence, with a known sample size. Pertinent conference proceedings published within this period were also eligible. Review articles, and case reports were excluded. There was no restriction based on study design, the age of participants and their gender, the place of the sampling, professional activities of study participants, ethnic group, or cultural behaviors. We have assessed all studies fulfilling these eligibility criteria and conducted on a sample of Burkina Faso’s population.

Study quality assessment was conducted based on the Grading of Recommendations Assessment, Development and Evaluation (GRADE) system [25]. Each study classified as high, moderate, low or very low quality of evidence. A study ID was assigned to any eligible study for summarizing purpose. Information collected included the location of sampling, the authors details, the study design, the year of sampling, the characteristics of the sample, participant age range, setting of study, sample size; diagnosis method, the number of positive and percentage. In some situation, information has been separated for details in case they addressed different population, location, gender or different date of sampling in the same study. Title and abstract screening, as well as full text data extraction were conducted by ML and crossed check by SO. If there where any disagreement, consensus was based on discussion whenever possible, if not decision was made by a third expert AMS.

### Data synthesis and analysis

The estimation of HBV prevalence was calculated based on the number of positive HBsAg subjects divided by the total number of subjects screened. Overall and sub-group specific prevalence estimates were conducted using the random effects meta-analysis model to take into account uncertainty in pooled estimates due to between study heterogeneity. Individual study proportions were assessed at 95% confident interval (CI) as well as pooled effect. Data were analyzed using the following formula in Additional file 1.

We conducted sub-analyses for the period in which studies samples were collected, according to the geographical location of the sampling, among different population sub-groups (pregnant women, HIV positive, general population, blood donors). To assess heterogeneity among study, we calculated the Index of inconsistency (*I*^2^) [26]. For comparison of proportion Chi-squared test was used and statistical significance level was set at *p* < 0.05.

### Test for publication bias

We assess possible publication bias by an Egger’s regression analysis and also graphically by a Funnel plot of all included studies.

### Ethical approval

This was a review of studies/data already published or available in the public domain, so the study did not require any ethical approval.

## Results

### General assessment

**Fig. 1:**
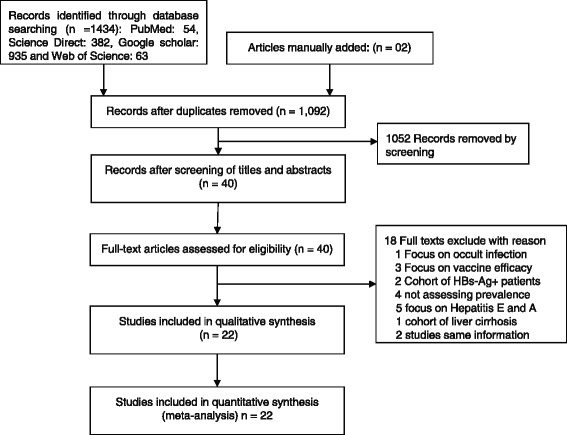
Flow chart of hepatitis B s-antigen seroprevalence studies included for systematic review and meta-analysis in Burkina Faso over a 20-years period (1996–2017)

**Fig. 2:**
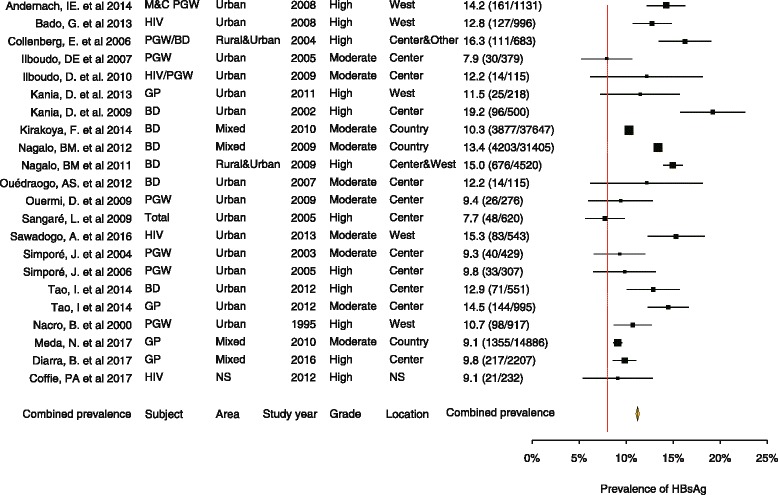
Forest plot of 22 prevalence studies and pooled prevalence using random effect model

A total of 1434 articles were identified through database search including 54 from PubMed, 382 from Science Direct, 935 from Google Scholar and 63 from the Web of Science. Two additional studies were manually added. After the exclusion of duplicate and irrelevant studies based on titles and abstracts screening, 40 full texts were retrieved and assessed in detail for eligibility criteria. Out of them, 22 studies met the eligibility criteria and were included in both qualitative and quantitative assessment. Figure 1 outlines the schematic flow diagram of the study detection and inclusion procedures. After full text analysis, 22 studies were summarized (Fig. 2) and included in the review. Report of prevalence was based on an aggregate sample size of 99,672 across all the 13-health regions of the country however mainly distributed between the two principal cities: Bobo-Dioulasso, the second main city with five (5) studies [27–31], and Ouagadougou, the capital city with eleven (11) studies [16, 17, 32–40]. Only two (2) published studies [41, 42] and one unpublished study (Meda N. et al. 2017 conference proceedings) where conducted on a countrywide basis. One (1) study included participants from the Boucle du Mouhoun region [32] and one (1) study included participants from the center-west region [15].

In term of population, seven (7) studies were conducted on first time voluntary blood donors [15, 16, 32–34, 41, 42]; Eight (8) studies were conducted on pregnant women [17, 27, 30, 32, 37–40]. A total of four (4) studies addressed HBV/HIV co-infection among HIV positive patients [28, 31, 37, 43], three (3) published studies [16, 29, 35] and one (1) unpublished study (Meda N. et al., 2017 conference proceeding) assessed the prevalence of HBV among general population. Children were not an important part of this evaluation, as none of the studies has addressed specifically children, however in 2 studies, age-adjusted analysis has given an overview of HBV infection in children and neonate population [17, 27]. Majority of studies (16/22) were conducted exclusively in urban areas while none of the study has addressed rural area alone. However three (3) studies have included in their analysis a subgroup of patients recruited from the rural areas [15, 27, 32]. About 36% (8/22) of studies were conducted before the implementation of HBV universal vaccination in the country. One (1) study has included patients in two different years in 2001, and in 2007 the implementation of the universal vaccine and ultimately published in 2014 [27].

**Fig. 3:**
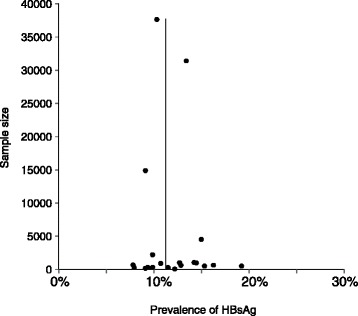
Funnel plot to assess publication bias

There was no evidence of publication bias with Egger’s regression analysis having a *p* value of 0.485. This was also illustrated graphically through a funnel plot of studies by a near symmetrical distribution of prevalence reported (Fig. 3). This means there is little effect of publication bias on the result of meta-analysis.

### Overall national estimations

**Fig. 4:**
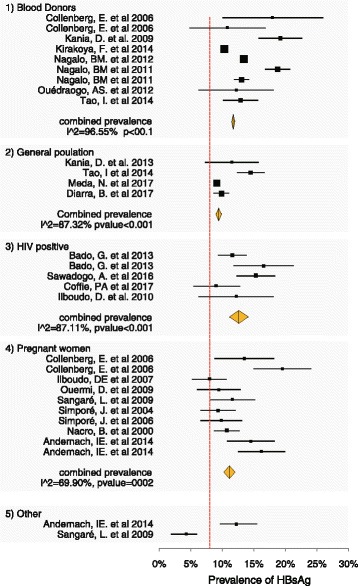
Trend of pooled prevalence over time regroups each 5-years

A total of 22 studies that included 99,672 participants were combined to estimate the national prevalence of hepatitis B chronic infection in Burkina Faso. The individual study prevalence ranged from 7.7% to 19.2% of HBV chronic carriers (Fig. 2). All studies have reported prevalence above or close to 8%, the threshold to define hepatitis B high transmission area [1, 3] except in the subgroup of neonates during their first 24 h of life with 4.24% of positive HBsAg. The nationwide-pooled prevalence estimate was 11.21% (95% CI: 11.01%–11.41%). However the analysis of between study heterogeneity has reported a strong evidence of difference with a heterogeneity index *I*^2^ = 94% and *p*-value < 0.001. Figure 3 presents each study prevalence estimate and the overall pooled prevalence obtained through a meta-analysis in a forest plot. We have adjusted the analysis to possible source of heterogeneity including sampling date, gender, location, and type of population to overcome this high heterogeneity (Fig. 4).

### Prevalence according to the sampling region

Studies conducted on countrywide basis have reported an estimate prevalence of 11.02% (95% CI: 10.81%–11.23%, I^2^ = 99%). However when studies were conducted in localized zone, the prevalence appeared lower in the central region 10.57% (95% CI: 9.85%-11.29%, I^2^ = 85%), and higher in the western part 12.69% (95% CI: 11.63%–13.74%, I^2^ = 38%). Few studies have been conducted in other regions, and meta-analysis has reported a prevalence of 14.66% (95% CI: 10.60–18.74, I^2^ = 0, p-value = 0.34) in semi-urban or rural area of the Boucle of Mouhoun region and 14.59% (95% CI: 13.57–15.62, I^2^ = 94%) in also a semi-urban area of the Center-west region of Koudougou. This indicates significant disparities in HBV prevalence between main cities and also between urban and rural areas. However, only few studies have investigated the prevalence of HBV carriage in the rural areas of Burkina Faso.

### Prevalence by study sampling period

**Fig. 5:**
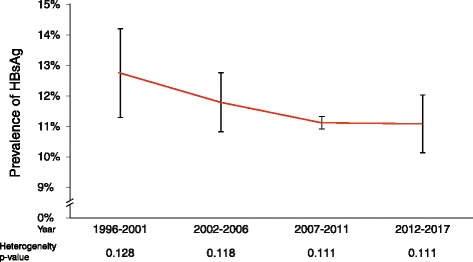
Forest plot analysis Subject type-specific Subgroup meta-analysis of HBV prevalence studies from Burkina Faso (1996–2017)

Some studies have collected their samples over different years as for Andernach et al. (2014) [27] which included samples collected in 2001 for mothers and children, and then in 2007 for pregnant women but all published in the same article in 2014. In this condition, we have split the sample for each year and analyzed them accordingly. To assess the temporal trend, we have placed the studies into 5-year interval categories. The meta-analysis reported a downward trend of prevalence over time (Fig. 5). Indeed the prevalence has significantly dropped from 12.80% (95% CI: 11.30–14.21) in 1996–2001 to 11.11% (95% CI: 10.14–12.03) in 2012–2017 (*p* = 0.0219). However the prevalence remained high above the 8% threshold of the WHO that defines a high transmission area.

### Prevalence among the general population

Only fours (4) studies have addressed the prevalence of HBV infection among the general population and included 18,306 participants. The estimate prevalence was lower than any of the specific groups. The prevalence was 9.41%(95% CI: 8.99–9.83, I^2^ = 87%).

### Prevalence among blood donors

A total of seven (7) studies have included first time blood donors in their samples. A total of 74,929 participants were included and represented 75% of the aggregated sample size. The meta-analysis showed that hepatitis B seroprevalence was high in blood donors with an estimate prevalence of 11.73% (95% CI: 11.50% -11.96, I^2^ = 96%).

### Prevalence among pregnant women

A total of eight (8) studies were conducted on pregnant women in the two main cities of the country. All studies were cross sectional and included a pooled sample size of 3810 pregnant women. The meta-analysis reported a pooled prevalence of 11.21% (95% CI: 10.22%–12.21%, I^2^ = 69%).

### Prevalence in among HIV positive individuals

A total of four (4) studies have assessed the prevalence of HBV among HIV positive individual in Ouagadougou and Bobo-Dioulasso. The estimate prevalence was 12.61% (95% CI: 11.11–14.11, I^2^ = 87%).

### Prevalence comparison between studies conducted in urban and rural areas

**Fig. 6:**
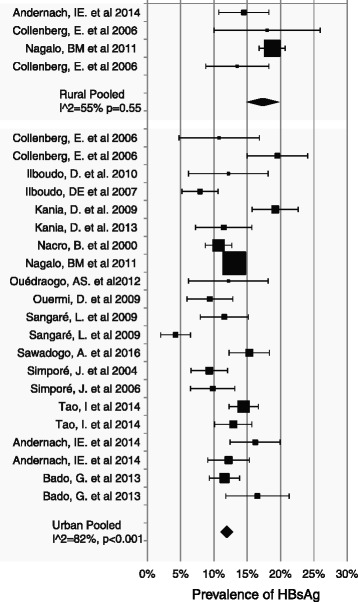
Forest plot analysis Area-specific Subgroup meta-analysis of HBV prevalence studies from Burkina Faso (1996–2017)

A total of sixteen (16) studies or sub-studies have addressed HBV prevalence across urban areas (Fig. 6). The estimate prevalence among the 11,150 participants was 11.93% (95% CI: 11.33–12.53, I^2^ = 82%). At same time only three (03) studies have addressed HBV prevalence across rural area, reporting an estimate prevalence of 17.35% (95% CI: 15.74–18.95, I^2^ = 55%) among a total of 2141 participants.

### Prevalence by comparison among different gender

Ten (10) studies have included a group of women in their analysis [17, 27, 28, 30, 32, 36–40]. Only one study has addressed distinctly men among HIV infected individuals [28]. The remaining studies have included both genders without adjustment in the analysis of prevalence. The prevalence among women was 11.30% (95% CI: 10.40–12.20, I^2^ = 63%) in an aggregated sample size of 4685 participants including pregnant and non-pregnant women. The remaining of studies have included both gender and the combined prevalence was 11.17% (95% CI: 10.97–11.37, I^2^ = 95%.

## Discussion

In Burkina Faso, the prevalence of HBV infection varies between 7.7% to 19.5% depending on the sampling population characteristics and period, the method of testing, and the design of the research study [33, 36]. However, all the studies conducted between 1996 and 2017 have reported a prevalence rate higher or closer to 8%, the threshold for the high transmission areas definition [44, 45]. According to the current review, about 1.8 millions and 2.1 millions cumulative individuals were infected in Burkina Faso in 2016, regardless of whether we considered the adjusted prevalence of 9.41% among the general population or the 11.21% estimate in the overall meta-analysis. HBsAg prevalence in Burkina Faso was estimated as 10.1% by WHO. It also supports the result of our study, that is high prevalence of HBsAg in Burkina Faso. This high burden represents a major challenge for the national health system that is already struggling with various infectious diseases like malaria, tuberculosis and acute respiratory infectious diseases [19]. The prevalence reported is in alignment with the 8.83% reported for the WHO region of Africa [3] and also with the previous evaluations listing Burkina Faso among the high transmission countries [27, 32, 35].

Urgent implementation of effective control measures, which can be integrated into a regional program and coordinated by an organism like the West African health organization (WAHO) through a concerted frame of activities would have better chance of success. Internally, curative and preventive actions should be promoted at all level, with access to effective and affordable diagnostics and treatments, to stop the cycle of transmission. In fact till now, the safe, highly effective and affordable medicine for chronic HBV management including tenofovir and entecavir are not yet available for the public since the WHO issued its first HBV management guidance in march 2015 [46]. Also even if HBV vaccination is available free in the EPI for children, it is still not covered outside EPI. Out-of-pocket payment for screening, and treatment medicines in the adult population is still a major obstacle. The reduction of financial barriers through the implementation of a universal health insurance system, and the availability of effective medicines could help to reduce the rate of transmission within the population. PROLIFICA study in The Gambia is one of effective way to introduction of treatment [47] .

The prevalence was higher in rural area compared to the urban city even though only few studies were conducted in the rural area and then the evidence that supports this difference weak. However with a low heterogeneity of studies conducted in the rural areas, it indicated a good evidence of a higher risk of infection among the rural population. Low economic status, low literacy rate, home birth, combined with high-risk behavior in the rural area like polygamous families, along with a limited availability of health services could explain this situation [19]. Evaluation of the actual prevalence of HBV in the rural area through well-designed studies could help draw a more complete picture of HBV infection burden in Burkina Faso. Beyond that, it is crucial to promote prevention methods, screening and treatment, safe sexual behaviors, raising awareness among rural populations and promote immunization among adult with negative HBsAg test results. Reducing financial barriers will be keys for the attainment of this objective.

The prevalence in Bobo-Dioulasso in the western region was significantly higher as compare to the prevalence in Ouagadougou in the central region. As Bobo-Dioulasso is a crossroad city in Burkina Faso leading to big cities including Abidjan in Ivory Coast and Bamako in Mali, this could be the reason why prevalence is higher in this city compared to Ouagadougou. Transport companies should be involved to raise awareness among travelers. This may be done through usage of prevention methods describe on posters and posted in stations and other gathering areas.

The prevalence of hepatitis B infection decreased significantly over time. This could be explained by the introduction of universal vaccination in 2006 in Burkina Faso as part of EPI and the promotion of diagnostic and vaccination among the adult population. Immunization may have by inference reduced the circulation of the virus among the whole population as studies published in the era (2007–2017) of post vaccine implementation have reported lower prevalence. However the prevalence remains high in the whole population. Control efforts should be scaled up countrywide and novel control approaches including screen-and-treat along with the vaccination could help reduce the burden of the disease in Burkina Faso.

In the general population the prevalence was higher than the 8% of the WHO definition of high transmission area [44], however it stayed significantly lower than any of the subpopulation prevalence. This may reports the actual prevalence of hepatitis B in Burkina Faso as it included even the group of children born after the implementation of HBV universal vaccination. However since the prevalence is already high, urgent actions should focus on controlling the disease in general population as well as in specific groups more at risk of the infection.

The prevalence of hepatitis infection was high among voluntary blood donors in Burkina Faso. The prevalence reported in this subpopulation was not representative of the whole population as children and women are not usual donors in Burkina Faso. The prevalence was comparable to data from Senegal with 12.1% donated blood contaminated [48]. This was far higher than data from Japan, in which it is reported between 1995 and 2000 a prevalence of only 0.63% contaminated donated blood supplies [49]. This difference describes the enormity of the problem of blood safety in Burkina Faso, and there is a need to take urgent actions to improve transfusion safety. This is particularly critical in Burkina Faso context as it raised a major concern about blood safety in a setting where blood transfusion is common mainly in children under 5 years old with anemia due to severe malaria, and which are at high risk of becoming chronic carriers if they were contaminated a that age [9, 10]. Control strategy will include a rigorous screening of blood supplies before transfusion and also a sensitization of blood donors with recent high-risk behavior to avoid blood donation as this have been suggested from the Japanese experience to be an effective strategy to reduce risk from acute infection cases still at the earlier stage of infection [50].

Hepatitis B prevalence among pregnant women remained very high and this should prompt a systematic screening of pregnant women during antenatal care visit, and treat those with high viral load to decrease the rate of antenatal mother to child transmission already high in the country [17]. Although the country has adopted universal immunization for HBV, it remains a combined pentavalent vaccine (hepatitis B, tetanus, diphtheria, whooping cough, and influenza type B) [19] only administered from 4 weeks onwards after birth, leaving children infected at birth and exposing them to more infection and to the development of chronic complications. EPI program should be adjusted to systematically administer hepatitis B immunoglobulin (HBIG) and vaccine to neonates within 24 h after birth focus on those born from HBsAg positive mothers. The vaccine is efficacious at 95% in preventing infection, the development of cirrhosis and HCC of hepatitis B origin and remains safe in children [51]. Indeed between 1990 and 2008, vaccine has decreased the prevalence of HBsAg from 14.6% in the1990 to less than 2% in a study conducted in the Gambia [52].

The prevalence among HIV positive individual was not so higher compare to other subgroup. As the prevalence of HIV is decreasing steadily [19], the problem will accordingly decrease. However urgent integrated measures should be implemented to manage this vulnerable group as they appear more at risk than the general population and then can represent an important source of contamination in the population. The effort toward the elimination of HIV should be maintained along with additional intervention for HBV infection.

All groups in the study, prevalence rates are high and more than 10%. Introduction of universal hepatitis B vaccination is expected to reduce the HBV horizontal infection and prevalence of HBV.

Political will and strong social awareness will be key to effectively tackling the problem in Burkina Faso. The purpose will be to solidify already used prevention methods like immunization and therefore make available the needed resources at each level to ensure dedicated activities are implemented adequately. It will be interesting if efforts deployed in the fight against HIV/AIDS could be replicated for HBV as this has allowed an important reduction of HIV prevalence from 10% in 1986 to almost 0.93% in 2014 in the country [53]. For this, a strong civil society action will be continuously required to keep health authorities alert to make adequate decisions, and also contribute to raise a wide social awareness of the problem at all level in the society. Health policymakers should make all efforts to make available affordable and effective screening methods, accessible and effective drugs for the management of those requiring treatment.

Also this review has revealed a paucity of data available on hepatitis B epidemiology in Burkina Faso, and there is therefore an urgent need to encourage research of solution well adapted to the context. Also it will be crucial to conduct study all over the country as almost 90% of the studies were conducted in the two main cities of the country while almost 80% of the population lives in the rural area [22].

In Addition studies were concentrated on adult population born before the establishment of the universal vaccination in Burkina Faso, so an evaluation of the prevalence of the infection in children born after the implementation of the policy will be interesting to assess the impact of the vaccination on the infection. It can also help predict the future trend of the disease in the country and the level of materno-fetal transmission. Further research in these areas would be needed to fully understand the dynamics of HBV burden in Burkina Faso.

## Limitations

This is a review of 22 studies conducted over 20 years period. They have included different sample sizes, and some of them included even a very small sample size. This has independently influenced the results. The quality of the test used, was not specified in some studies and the procedure of sample selection not detailed. Also the usage of multiple generation of test with possible evolution in the sensitivity and specificity may have influenced the prevalence reported in each of them. This has shown a high evidence of heterogeneity statistically supported by an index of heterogeneity of 94%. In subpopulation analysis according, to possible source of heterogeneity, only the setting of the sampling (urban and rural) are formally proven to influence the prevalence of hepatitis B infection. Still, this evidence remains very weak because only few studies included in this review have involved participants from the rural area and majority of the studies came from urban cities, Ouagadougou and Bobo-Dioulasso. However, the prevalence reported could still guide policymaker for the allocation of resources before well-designed studies will close the gap of information to eliminate the disease.

## Conclusions

The prevalence of hepatitis B burden is high among Burkinabè population as describe by high prevalence rate at all levels of the population. This is a preventable disease and an effective program of immunization was implemented two decades ago. However a large number of people infected with HBV were born before the implementation of the hepatitis B universal vaccination. Intervention that targets the older population along with the current immunization program have the potential to help reach the target of eliminating hepatitis B as a major public health issue. Hepatitis B infection management should be of multi-disciplinary approach involving politics, patients, health care workers and a strong civil society. National program will have to introduce hepatitis B infection among priority diseases, and integrate universal immunization for children starting in their first 24 h, a screen and treat strategy for already infected individual and a special attention to prevention and raising global awareness. Experience from HIV control program should be capitalized for viral hepatitis across the country.

## Additional file

Additional file 1: Method of meta-analysis. The document contains the calculation of meta-analysis. (PDF 268 kb)

## Abbreviations

AIDS: acquired immune deficiency syndrome
CI: confident interval
EPI: expanded program on immunization
GDP: gross domestic product
HBeAg: hepatitis B e antigen
HBIG: hepatitis B immunoglobulin
HBV: hepatitis B virus
HCC: hepatocellular carcinoma
HIV: human immunodeficiency virus
ID: identification
Mesh: medical subject headings
PRISMA: preferred reporting items for systematic review and meta-analysis
USD: U.S. dollar
WAHO: West African health organization
WHO: World health organization
