# Supplementary material for: High prevalence of hepatitis B infections in Burkina Faso (1996–2017): a systematic review with meta-analysis of epidemiological studies
Source: BMC Public Health. 2018 Apr 25;18:551. doi: 10.1186/s12889-018-5432-7 (PMC5921387; doi:10.1186/s12889-018-5432-7)
Supplement: Supplementary file 1 — Method of meta-analysis. The document contains the calculation of meta-analysis. (PDF 268 kb) [file 12889_2018_5432_MOESM1_ESM.pdf]

Appendix: Method of meta-analysis

Variance of the studies and their weights in fixed model are given as follow[Ref]: $v_i = [p_i (1 - p_i)]/ n_i$, and $w_i = 1 / v_i$, where i, n_i and p_i denote index, sample size and prevalence of the study respectively. Using average weight and its variance as follow, $\bar{w} = \Sigma_i w_i / k$, and $s_w^2 = (\Sigma_i w_i^2 - k\bar{w}^2) / (k - 1)$, we calculated statistic U and $\hat{\tau}$ as $U = (k - 1) \{\bar{w} - s_w^2/(k\bar{w})\}$ and $\hat{\tau} = \max\{0, (Q - (k - 1))/U\}$. Then, weight in random-effect model and pooled prevalence were obtained by $w_i^* = 1 / (\hat{\tau}^2 + v_i)$, and $\hat{p} = \Sigma_i w_i^* p_i / \Sigma_i w_i^*$.

To assess heterogeneity among study, we calculated the Index of inconsistency (I^2) [24] as $Q = \Sigma_i w_i (p_i - \hat{p})^2$ and $I^2 = 100*\{Q - (k - 1)\}/Q$.

Ref. Tango T. Introduction to Meta-analysis (in Japanese), Tokyo:Asakura-shoten; 2016
